# Metabolomic Changes in Plasma of Preterminal Stage of Rhesus Nonhuman Primates Exposed to Lethal Dose of Radiation

**DOI:** 10.3390/metabo14010018

**Published:** 2023-12-27

**Authors:** Alana D. Carpenter, Oluseyi O. Fatanmi, Stephen Y. Wise, Sarah A. Petrus, John B. Tyburski, Amrita K. Cheema, Vijay K. Singh

**Affiliations:** 1 Division of Radioprotectants, Department of Pharmacology and Molecular Therapeutics, F. Edward Hébert School of Medicine, Uniformed Services University of the Health Sciences, Bethesda, MD 20814, USA; alana.carpenter.ctr@usuhs.edu (A.D.C.);; 2 Armed Forces Radiobiology Research Institute, Uniformed Services University of the Health Sciences, Bethesda, MD 20814, USA; 3 Nelson Scientific Labs, LLC, Potomac, MD 20854, USA; 4 Department of Oncology, Lombardi Comprehensive Cancer Center, Georgetown University Medical Center, Washington, DC 20057, USA; 5 Department of Biochemistry, Molecular and Cellular Biology, Georgetown University Medical Center, Washington, DC 20057, USA

**Keywords:** biomarkers, metabolomics, nonhuman primates, preterminal, total-body irradiation

## Abstract

Ionizing radiation exposure is known to induce molecular and cellular injury, inflicting a cascade of potentially catastrophic events leading to tissue and organ damage. Metabolomic analysis allows for the identification and quantification of small molecules downstream of genomic changes induced by radiation exposure. We aimed to characterize metabolomic changes that underscore the prefinal stage of lethally irradiated rhesus nonhuman primates (NHPs). Peripheral blood was drawn at baseline, post-exposure, as well as at the preterminal stage in NHPs (immediately prior to death in moribund NHPs) that did not survive exposure with 7.2 Gy total-body radiation (LD_70/60_). Herein, we analyzed global metabolomic changes using ultra-performance liquid chromatography (UPLC) quadrupole time-of-flight mass spectrometry (QTOF-MS) in plasma samples of NHPs collected at various timepoints in relation to irradiation. The overall goal was to identify metabolic shifts present immediately prior to death. Our findings showed radiation induced significant time-dependent metabolic perturbations when compared to pre-irradiation profiles, particularly in glycerophospholipid metabolism and steroid hormone biosynthesis and metabolism pathways. These findings provide valuable insights for identifying biomarkers for lethality, which may be helpful for triage during a mass casualty scenario.

## 1. Introduction

The threat of a nuclear or radiological event is a serious concern for all government agencies involved in public health preparedness and national security, as well as for the military personnel [1,2]. Management of such nuclear and radiological causalities as a result of nuclear power plant accidents or safety failures, natural calamities, terrorist attacks, or military conflicts will need medical interventions [3,4,5]. The BioShield legislation, signed into law on 21 July 2004, provides new tools to improve preparedness for Americans against a chemical, biological, radiological, or nuclear (CBRN) threat [6,7,8]. In the event of a nuclear or radiological scenario, medical care would be needed to treat radiation-exposed victims developing acute radiation syndrome (ARS) [9,10,11]. Acute radiation injury occurs at total-body doses above 1 Gy, with symptoms growing in severity as the level of radiation exposure increases [9]. A dose up to 6 Gy for human is characterized by the loss of hematopoietic cell regenerative ability, resulting in hematopoietic ARS (H-ARS). 

Biomarkers are important for assessing the dose of radiation exposure as well as for the development of radiation medical countermeasures (MCMs). In fact, the identification of efficacy-based biomarkers is required by the United States Food and Drug Administration (US FDA) under the Animal Rule for conversion of dose from animals to humans for approval of any MCM [12,13]. Ionizing radiation is known to induce a cascade of changes that can have serious deleterious implications for key metabolic processes and such metabolomic changes can be used as radiation injury biomarkers [14,15,16]. Metabolomics is an emerging discipline and metabolites are products of biological cascades of proteomic and genomic processes, and the presence of such metabolites is linked with the individual’s tissue/cell phenotype, making them ideal biomarkers for injury or disease progression. These metabolite biomarkers are also useful due to their comparative ease of identification and validation in biological fluids collected without major invasive procedures. Both untargeted (global) as well as targeted (quantitative phase) metabolomic techniques have been widely used in various settings of radiation injuries [15,16]. Sometimes the appearance of H-ARS as a differential diagnosis is detected late and not until the preterminal stage, as in the Litvinenko case with radionuclide incorporation [17,18,19]. Similar was the situation in the large-scale exposure event of Goiania, Brazil, which occurred due to a misused orphan radioactive source [20,21]. Thus, the identification of biomarkers for preterminal stage is important.

Nonhuman primates (NHPs) are the gold standard of animal models acceptable to the US FDA for advanced development of biomarkers and countermeasures to evaluate translational feasibility for humans [22]. Although a large number of studies have been performed that assessed concentrations of metabolic profiles in blood plasma/serum and tissue samples collected from NHPs exposed to various types and doses of radiation [15,16], no study has investigated the metabolic profiles in samples collected from radiation-exposed moribund (preterminal) NHPs. There is interest to know whether changes in biofluids can serve as biomarkers for prefinal deteriorated health status [23]. The objective of this study is to identify metabolite biomarkers for moribundity which can be used for triage of exposed victims. To understand this, we studied the altered metabolites in peripheral blood plasma samples collected from moribund NHPs that did not survive after lethal radiation exposure. In brief, the purpose of this study was to characterize the metabolomic changes in blood plasma collected from NHPs following exposure to 7.2 Gy (LD_70/60_) total-body ionizing radiation with particular attention on how the metabolite profiles differed from those samples collected at pre-exposure and early post-exposure timepoints. The preterminal samples were collected immediately prior to euthanasia of moribund animals, which ultimately provided insight into the preterminal phenotype of moribund animals. These data are of particular interest because they allow for the identification of potential patterns in dysregulated metabolites that may be used as biomarkers during triage after a mass casualty scenario. To our knowledge, this is the first study evaluating the metabolic profiles in irradiated samples collected from moribund (preterminal) NHPs to identify the metabolites significantly altered just before death.

## 2. Materials and Methods

### 2.1. Experimental Design

**Figure 1:**
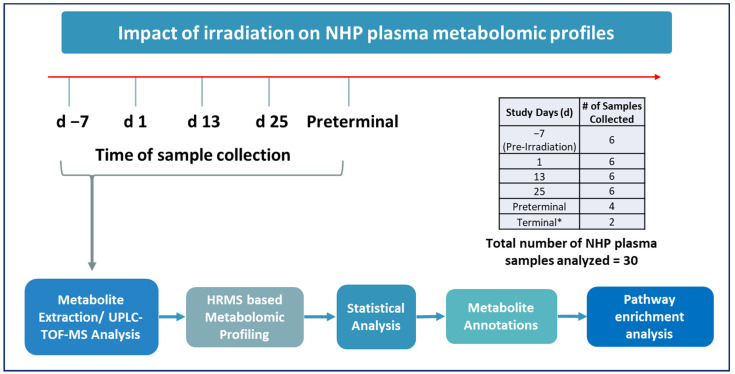
Experimental design to assess changes in plasma metabolic profiles in NHPs exposed to 7.2 Gy TBI from samples collected pre-irradiation (day (d) -7), d 1, d 13, d 25, or immediately prior to death (preterminal). # Total number of samples collected at each timepoint for metabolomic analysis. * Since the terminal group consisted of only two samples, it was not included in any comparisons.

The primary objective of this study was to identify metabolic changes in plasma samples collected from NHPs prior to and following exposure to 7.2 Gy TBI as well as in samples collected from preterminal animals. Preterminal samples have been compared to samples from animals prior to irradiation and at several timepoints after irradiation. Details of the experimental design can be viewed in Figure 1. 

### 2.2. Animals

Fourteen male NHPs (*Macaca mulatta*, Chinese sub-strain) between the ages of 3.0 to 5.3 years and weighing 3.89 to 6.34 kg were procured from the National Institutes of Health Animal Center located in Poolesville, MD. These animals were housed at the Armed Forces Radiobiology Research Institute (AFRRI) vivarium, a facility accredited by the Association for Assessment and Accreditation of Laboratory Animal Care (AAALAC)-International. Prior to initiation of the study, the animals underwent a mandatory quarantine for seven weeks. Animals were housed individually to prevent possible conflict injuries; however, cage dividers were present, which allowed for animals to interact socially. A primate diet (Teklad T.2050 diet; Harlan Laboratories Inc., Madison, WI, USA) was provided twice per day, with at least 6 h between feedings (each animal was provided 4 biscuits at 7:00 a.m. and 2:00 p.m.). Drinking water was provided *ad libitum*. The study design and animal procedures were reviewed and approved by The Institutional Animal Care and Use Committee and the Department of Defense Animal Care and Use Review Office (ACURO). All animal procedures strictly adhered to the Guide for the Care and Use of Laboratory Animals throughout this study as described earlier [24,25].

### 2.3. Irradiation

Animals were grouped in pairs for radiation exposure; pairing was based on comparability of their abdominal lateral separation measurements (±1 cm). These measurements were taken using a digital caliper at the core of the abdomen. Animals with an abdominal measurement that was not within 1 cm of another animal’s measurements were irradiated individually. Beginning approximately 12–18 h prior to radiation exposure, all NHPs were fasted to reduce the risk of radiation-induced vomiting. About 15 min prior to irradiation, animals were restrained using the squeeze-back mechanism of their cage and sedated with 10–15 mg/kg of ketamine hydrochloride (100 mg/mL) administered intramuscularly (*im*). The sedated animals were placed in custom-made Plexiglas restraint boxes and secured using ropes that anchored their arms and legs in place. NHPs were given a booster (0.1–0.3 mL *im*) of ketamine if needed immediately prior to initiating the radiation exposure to reduce any potential movement. The NHP pair was then placed facing opposite directions on the irradiation platform, and were exposed to cobalt-60 total-body gamma radiation at a dose of 7.2 Gy and a dose rate of 0.6 Gy/min. After radiation exposure, NHPs were returned to their home cages and monitored closely by study staff until they completely recovered from sedation. Additional details of total-body irradiation and dosimetry are given in earlier publications [26,27]. Animals were exposed to radiation between 8:00 a.m. and 12:00 noon.

### 2.4. Cage-Side Animal Observations

Cage-side animal observations were performed at least twice a day in the morning and the afternoon for the quarantine and study periods. Beginning on the day of irradiation, clinical observations were recorded and reported to the study director and veterinarian once per day. Between days 10 to 20 post-irradiation, animals were observed three times a day between 6 and 8 h apart. Animals that were deemed moribund using criteria listed on the approved protocol were scheduled for euthanasia based on the veterinarian’s discretion. Several parameters were used as guidelines for moribundity including inappetence, minimal, or no response to stimuli, severe anemia, weakness, etc. [25]. An on-call veterinary technician/veterinarian was available 24 h a day in the event of an emergency situation [28]. 

### 2.5. Blood Sample Collection

Plasma fraction was enriched from whole blood samples collected on days -7, 1, 13, and 25, or immediately prior to euthanasia (preterminal). Blood was collected from a peripheral vessel (either saphenous or cephalic vein) as described earlier [29]. One ml of blood was collected with a three mL disposable luer-lock syringe with a 25-gauge needle into an ethylenediaminetetraacetic acid (EDTA) tube and plasma was separated by centrifugation.

### 2.6. Euthanasia

The study period was for 60 days, however, due to the radiation dose used (7.2 Gy TBI, LD_70/60_), a few animals became moribund during the course of the study and were deemed candidates for early euthanasia to minimize unnecessary pain and suffering to the animal. The animals were euthanized according to the American Veterinary Medical Association (AVMA) guidelines as described earlier [30,31]. In brief, for euthanasia, animals were sedated with ketamine hydrochloride (5–15 mg/kg, *im*) injection, then euthanized by sodium pentobarbital intravenously (>100 mg/kg, Euthasol, Virbac AH, Inc., Fort Worth, TX, USA). Death was confirmed by cessation of pulse, heartbeat, and breathing.

### 2.7. Sample Preparation for LC-MS Analysis

For metabolomics analysis, a plasma volume of 25 µL was obtained from each sample and transferred to a newly labeled vial. To each aliquot, a volume of 75 µL of extraction solution (35% water, 25% methanol, 40% isopropanol, 0.001% Debrisoquine, 0.005% 4-nitrobenzoic acid) was added. Samples were then vortexed and incubated for 20 min on ice. A volume of 100 µL of chilled acetonitrile was then added to each sample. Samples were vortexed and kept at −20 °C for 15 min. Next, samples were centrifuged at 15,500× *g* at 4 °C for 20 min. The supernatant from centrifuged samples was transferred to glass vials. To prevent bias, the sample queue was randomized before data acquisition.

### 2.8. Plasma Metabolomics Using UPLC QTOF Analysis

Metabolite extraction was performed as described above and previously [32]. The sample extracts were resolved on an Acquity UPLC coupled to a Xevo G2 QTOF-MS (Waters Corporation, Milford, MA, USA). The volume of each sample was 2 μL. For metabolomics acquisition, the sample was injected onto an Acquity UPLC BEH C18, 130 Å, 1.7 µm, 2.1 mm × 50 mm column maintained at 40 °C. For lipidomics acquisition, the sample was injected onto a CSH C18, 130 Å, 1.7 µm, 2.1 mm × 100 mm column maintained at 65 °C. The LC solvents used were as followed: (A) 100% water with 0.1% formic acid, (B) 100% ACN with 0.1% formic acid, and (C) 100% isopropanol with 0.1% formic acid and 10 mM ammonium formate. The metabolomics gradient with a flow rate of 0.5 m/min was set as followed: initial—98% A, 2% B; 0.5 min—98% A, 2% B; 4.0 min—40% A, 60% B; 8.0 min—2% A, 98% B; 9.0 min—2% B, 98% D; 9.5 min—11.2% B, 88.2% C; 11.0 min—11% B, 88.2% C; 11.5 min—50% A, 50% B; 12.0 min—98% A, 2% B, 13.0 min—98% A, 2% B. The lipidomics solvents were the same as previously mentioned with the addition of 10 mM ammonium formate. The gradient had a flow rate of 0.45 m/min and was run set as followed: initial—30% A, 34% B, 36% C; 0.5 min—30% A, 34% B, 36% C; 8.0 min—10% B, 90% C; 8.5 min—10% B, 90% C; 9.0 min—30% A, 34% B, 36% C; and 11.0 min—30% A, 34% B, 36% C [32].

The column eluent was introduced into the QTOF MS that was operating in either positive or negative modes by electrospray ionization [33]. While the positive mode had a capillary voltage of 3.00 kV and a sampling cone voltage of 30 V, the negative mode had a capillary voltage of 2.00 kV and had a sampling cone voltage of 30 V. The desolvation temperature was set to 500 °C, and the desolvation gas flow was set to 1000 L/h. The source temperature was 120 °C, while the cone gas flow was 25 L/h. The sensitivity mode was used to acquire data with a scan time of 0.300 s and an interscan time of 0.014 s. Accurate mass was maintained by infusing leucine enkephalin (556.2771 *m*/*z*) in 50% aqueous acetonitrile (2.0 ng/mL) at a rate of 20 µL/min via the Lockspray interface every 10 s. Data was acquired in Centroid mode with a 50.0 to 1200.0 *m*/*z* mass range for ToF MS scanning [27]. The pooled QC was injected every 10 samples to monitor any shifts in retention time and intensities.

### 2.9. Statistical Analysis

In this study, we compared metabolite abundance that was represented by normalized intensity units. Data obtained from electrospray positive and negative modes were analyzed separately after internal standard and Quality Control-based Robust LOESS Signal Correction (QCRLSC) normalization. The data matrix had some inherent limitations with respect to sample availability across different time points with some, but not all, having repeat measures. To address this, we applied both independent (unpaired) and dependent (paired) statistical tests, with results detailed in Supplementary Tables S1 and S2. We considered a *p*-value of less than 0.05 as statistically significant. For unpaired comparisons, we employed the Mann–Whitney U test, a nonparametric technique, to evaluate the distributional equality between two distinct populations. This test is especially pertinent when the data does not follow normal distribution. For paired comparisons, the Wilcoxon signed-rank test was utilized. Both of these tests are appropriate for nonparametric data analysis. To address the issue of multiple comparisons, which heightened the likelihood of false positives, we applied the False Discovery Rate (FDR) method to adjust *p*-values. This approach is less stringent than traditional family-wise error rate corrections and is more apt for studies with a substantial number of tests. Additionally, our study incorporated the Kyoto Encyclopedia of Genes and Genomes (KEGG) pathway analysis. This method maps molecules from our dataset onto KEGG’s curated biological pathways, providing insight into the biological processes and pathways that are active, altered, or disrupted in our study conditions. KEGG pathway analysis helped contextualize molecular data within a biological framework, offering a comprehensive view of the systemic functions affected by the studied metabolites. Taken together, this approach, combining statistical rigor with biological pathway analysis, ensures a thorough and nuanced interpretation of our findings, considering the variability in sample availability and providing deeper insights into the biological significance of our results.

## 3. Results

**Figure 2:**
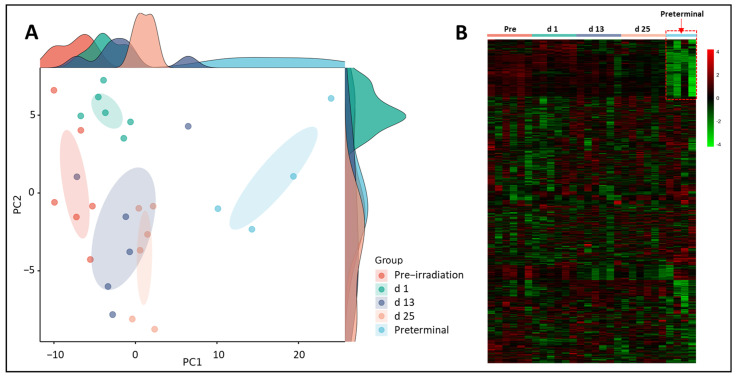
Principal Component Analysis (PCA) plot (Panel **A**) and heat map (Panel **B**) show radiation induced robust changes in metabolic profiles in pre-irradiation, day (d) 1, d 13, d 25, or preterminal samples. Of note, the plasma metabolic profiles of the preterminal samples are strikingly different compared to other study groups. The highlighted metabolites are discussed in Table 1, Table 2 and Table 3.

Initially, we performed binary comparisons to assess the effects of radiation on NHP plasma metabolic profiles by comparing samples collected at days 1, 13, or 25 with day -7 (pre-irradiation). Separately, we also compared metabolic profiles of and samples collected at days 1, 13, or 25 with preterminal to assess the metabolic signatures associated with the preterminal state. A Principal Component Analysis (PCA) score plot and a heat map were constructed to view overall differences among groups (Figure 2). The PCA scores for the preterminal samples are distinct from all other scores along the PC1 dimension, which represents the most important directionality within the data matrix. Although dominated by the preterminal scores’ separation, there is also apparent separation along PC1 of the day 25 scores from a cluster of both the day 13 and day 1 scores, which itself separates to some extent from the pre-irradiation sample scores. Interestingly, we found along the PC2 dimension, day 1 scores mostly separated from those of day 13 and day 25, suggesting a noticeable but transient early radiation response in the plasma metabolome was present that warranted further investigation.

### 3.1. Putative Biochemical Changes Associated with Ionizing Radiation Exposure

**Figure 3:**
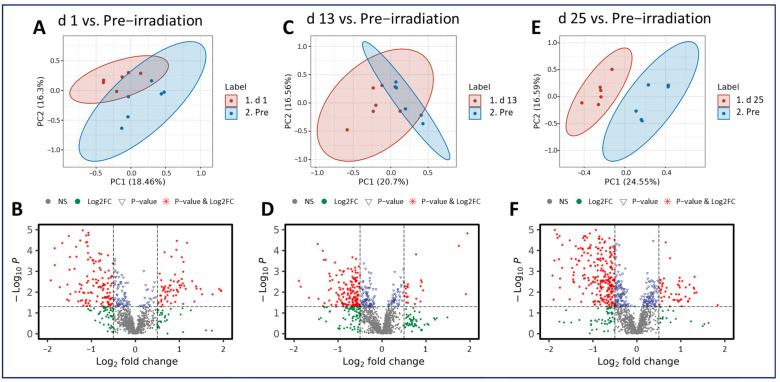
PCA and volcano plots illustrating metabolic disruptions caused by radiation at days (d) 1 (**A**,**B**), 13 (**C**,**D**), and 25 (**E**,**F**), respectively.

Radiation exposure induced metabolic shifts that were increasingly downregulated as the study progressed. Interestingly, when comparing across all three post-irradiation timepoints (day 1, 13, or 25) to the pre-irradiation timepoint (day -7), the majority of the metabolites analyzed were downregulated, which was demonstrated in the volcano plots (Figure 3). These included PC (20:2/18:0) and PE (22:6/18:2) (Table 1). This pairwise stratification of the pre-irradiation timepoint to the post-irradiation timepoints supports the notion that metabolic perturbations following a single IR exposure change substantially over time. Significant metabolites were detected between all comparisons, but the greatest difference was noted between the pre-irradiation time point and day 25. The features that were altered significantly in each comparison and that were matched in the Metlin database search were shown as clusters according to their involvements or associations. Prominent among these is the involvement in or association with the alpha-linolenic acid, choline, and glycerophospholipid metabolic pathways. Additional information on the notable metabolites and related pathways for these comparisons can be viewed in Supplementary Figures S1–S3 and Supplementary Tables S3–S5.

### 3.2. Putative Biochemical Changes Associated with the Preterminal State after Ionizing Radiation Exposure

As was seen in the pre-irradiation comparisons, radiation-induced metabolic perturbations were downregulated as the study progressed. However, the comparison between the preterminal timepoint to the post-irradiation timepoints revealed the majority of metabolites in these comparisons were upregulated. 

**Figure 4:**
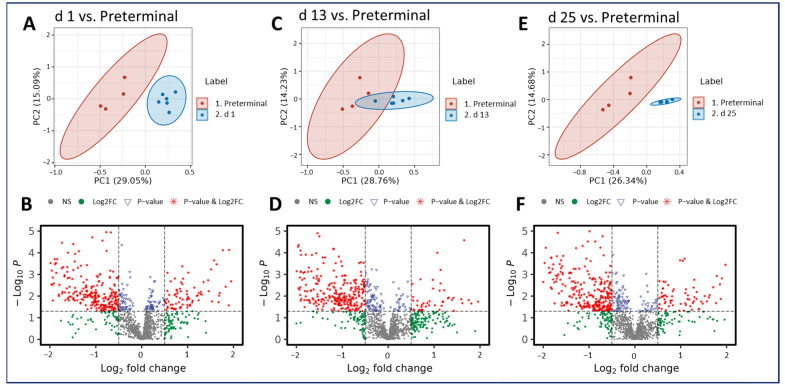
PCA and volcano plots demonstrating metabolic alterations when comparing the preterminal stage to the post-irradiation stages at day (d): d 1 (**A**,**B**), d 13 (**C**,**D**), and d 25 (**E**,**F**), respectively.

Notably, there are more statistically significant disruptions in the plasma samples of preterminal NHPs compared with those collected at the day 1, 13, and 25 timepoints (Figure 4). However, the pre-irradiation vs. preterminal comparison revealed the greatest number of significant metabolites, and the majority were downregulated. Three metabolites putatively involved in steroidal hormone biosynthesis appeared in greater abundance in the preterminal NHP samples compared with all other post-irradiation timepoints tested, with fold changes ranging from 1.47 to 9.49. These upregulated metabolites include 5alpha-pregnane-3,20-dione, 17alpha-hydroxypregnenolone, and allopregnanolone, and results are summarized in Table 2. Additionally, a number of plasma metabolites were detected at lower abundances in preterminal NHP samples compared with samples collected at all other timepoints. Four metabolites including LysoPA (18:2), PE (22:6/18:2), androsterone, and testosterone were consistently downregulated and were summarized in Table 3. Tandem mass spectrometry using collision induced dissociation (CID) was performed for unambiguous identification of a subset of metabolites by matching MS/MS spectra against the NIST spectral database. The subset of metabolites for which the CID fragmentations confirmed identities were listed in Table 1, Table 2 and Table 3, and the putative annotations were included in Supplementary Tables S1 and S2, as well as Supplementary Figures S1–S6. Fold-changes for these seven metabolites ranged from 0.678 to 0.176, representing percent decreases from 32.2 to 82.4, respectively. Pathway enrichment analysis suggests these metabolites seem to be involved in choline, alpha-linoleate, glycerophospholipid, and steroid hormone synthesis and metabolism. Additional information on the notable metabolites and related pathways can be viewed in Supplementary Figures S4–S6 and Supplementary Tables S6–S8. Additional set of metabolites putatively associated with a variety of metabolic pathways and mechanisms were significantly altered when examining pairwise the preterminal and other timepoints.

## 4. Discussion

Biomarkers, or biological markers, are quantifiable biological characteristics that can be used as a surrogate to assess overall health of an individual at a certain point in time and can include proteins, metabolites, cytokines, etc. Biomarkers are of particular interest in radiation biology, as they can be used to assess the extent of radiation exposure, predict lethality, and to assess drug efficacy in the development of radiation countermeasures. Given radiation is known to induce damage at the genetic level, the expression of these changes can be viewed downstream in the form of metabolomic changes. Identifying the metabolic pathways involved in the cellular response to radiation exposure is therefore essential for evaluating the overall health of an individual and for the development of radiation countermeasures. And, this will potentially allow for these particular metabolites and pathways to be targeted for the development of radiation countermeasures [34]. Metabolomic changes induced by acute radiation exposure have been well-documented in our previous studies; we have investigated metabolic changes in both serum and tissue samples in murine [35,36,37] and NHP [38,39,40,41] models that were exposed to ^60^Co total-body γ-radiation, and also in NHPs exposed to partial-body LINAC-derived X-ray exposure [42]. In these studies, various radiation MCMs that were being developed for prophylactic or mitigative use were administered to assess the effects on metabolomics, including Ex-Rad [27], BIO 300 [32,43], amifostine [35,36,37], and GT3 [44,45]. The Ex-Rad [27] and GT3 [42,44] studies were performed with an NHP model, while the amifostine [35,36,37] studies were performed in a murine model. Interestingly, we have performed two additional metabolomic studies with 7.2 Gy TBI using NHP model, one assessing irradiation-induced changes in serum and the other assessing irradiation-induced changes in tissue. These studies did not attempt to classify biomarkers unique to preterminal NHPs; however, the results of these studies confirmed distinct differences in metabolomic profiles induced by radiation, particularly in acylcarnitines [39,41]. However, this is the first study where we have attempted to identify metabolic shifts presented immediately prior to death in irradiated, preterminal animals. 

In this study, fourteen male NHPs were exposed to 7.2 Gy total-body gamma irradiation and were monitored for 60 days post-irradiation. The main goal of this study was to characterize the metabolomic perturbations involved in the preterminal state following IR exposure. Plasma samples were collected throughout the course of the study and were analyzed to assess changes in metabolomic profiles as a function of time in the survivor and pre-terminal cohort. As this study used a lethal dose of radiation (LD_70/60_), a few NHPs became moribund and were deemed candidates for early euthanasia. In an effort to catalog the preterminal phenotype of NHPs on the verge of death, blood samples were taken from these animals immediately prior to euthanasia. Comparisons were performed to assess any significant changes in pre-irradiation or preterminal samples. 

As expected, radiation induced a cascade of significant metabolic changes that affected several key metabolic pathways. The alpha-linolenic acid, choline, and glycerophospholipid metabolic pathways were significantly altered by radiation exposure when comparing post-irradiation samples to pre-irradiation samples. In preterminal samples, a greater degree of metabolic dysregulation was observed in comparison to all other timepoints tested. Both the glycerophospholipid metabolism and steroid hormone and biosynthesis pathways were significantly dysregulated in preterminal animals. Interestingly, glycerophospholipid metabolism was found to be dysregulated both in post-irradiation and preterminal samples. This pathway is of particular interest, as radiation is known to induce lipid peroxidation and dyslipidemia resulting in cellular damage [46]. Both the glycerophospholipid metabolism pathway and the steroid hormone and biosynthesis pathway are involved in inflammatory response, and several other studies demonstrate dysregulation in these pathways is induced by radiation exposure [27,47,48]. Three steroid hormones, 5alpha-pregnane-3,20-dione, 17alpha-hydroxypregnenolone, and allopregnanolone, were found to be significantly upregulated in preterminal NHPs. The steroid hormone biosynthesis pathway seems to be particularly sensitive to radiation exposure, which may indicate dysregulation to this specific pathway may increase lethality in irradiated animals [27,37,47]. These results suggest early perturbations of these specific pathways and metabolites may be representative of the preterminal state of lethally irradiated NHPs.

Evidence from this analysis, while exploratory, strongly supports the notion plasma samples from preterminal NHPs exhibit a number of significant metabolomic alterations or disruptions that can be used as preemptive indicators of radiation lethality. Based on putative metabolic feature identities, the same or similar pathways were disrupted following irradiation as were disrupted at the preterminal stage. To the best of our knowledge, this is the first study investigating the metabolic profiles in irradiated NHP samples collected from moribund (preterminal) NHPs to identify the metabolites significantly altered just prior to death. Additionally, another study is being conducted using similar preterminal samples from a large number of NHPs irradiated with more than one dose of cobalt-60 gamma-radiation. Continued research into the preterminal state of moribund NHPs is needed to further identify metabolites and pathways that can be targeted for the development of various therapeutic strategies to treat ARS. Additionally, next steps also include performing tandem mass spectrometry of both the unknown compound in-sample and a known standard compound against the NIST spectral database to confirm compound identities. 

## Figures and Tables

**Table 1:** Downregulated plasma metabolites when comparing pre-irradiation samples to day (d): d 1, d 13, and d 25 samples.

				*p*-Value		
Compound	Comparison	FC	Log2(FC)	WSR Test	MWU Test	CID Fragments
PC (20:2/18:0)	d 1 vs. pre-irradiation	0.62122	−0.68682	<0.05	0.001	858.6165 798.5977 307.2622 283.2641
	d 13 vs. pre-irradiation	0.87591	−0.19114546	0.24	0.292	
	d 25 vs. pre-irradiation	0.79989	−0.32212648	<0.05	0.026	
PE (22:6/18:2)	d 1 vs. pre-irradiation	0.59364	−0.75234	<0.05	0.005	786.5251 327.2357 283.2443 279.2334
	d 13 vs. pre-irradiation	0.70821	−0.49775088	0.24	0.061	
	d 25 vs. pre-irradiation	0.49562	−1.01269369	<0.05	0.003	

FC: fold change, WSR: Wilcoxon Signed-Rank test, MWU: Mann–Whitney U test.

**Table 2:** Upregulated plasma metabolites in preterminal samples compared to day (d): d 1, d 13, and d 25 samples.

				*p*-Value		
Compound	Comparison	FC	Log2(FC)	WSR Test	MWU Test	CID Fragments
5alpha-Pregnane-3,20-dione	d 1 vs. preterminal	1.8672	0.900876466	0.01	0.002	315.2558 278.9958 266.9861 228.9908 315.2539 297.2443 278.9866 266.9792
	d 13 vs. preterminal	1.5528	0.634872023	0.02	0.017	
	d 25 vs. preterminal	1.9819	0.986884171	0.01	<0.001	
17alpha-Hydroxypregnenolone	d 1 vs. preterminal	9.4901	3.246423289	0.01	<0.001	297.1469 282.1202 331.1852 315.1572 297.1487 282.1252 189.0946 135.0848 125.0597
	d 13 vs. preterminal	2.4951	1.319097638	0.01	0.012	
	d 25 vs. preterminal	3.7344	1.900876466	0.01	0.002	
Allopregnanolone	d 1 vs. preterminal	1.715	0.778208576	0.01	0.004	319.2986 281.6630 272.6601 184.0767 148.5324 319.2920 281.6631 272.6624 184.0746 145.5324
	d 13 vs. preterminal	1.4744	0.560127976	0.07	0.04	
	d 25 vs. preterminal	1.6643	0.734915511	0.04	0.015	

FC: fold change, WSR: Wilcoxon Signed-Rank test, MWU: Mann–Whitney U test.

**Table 3:** Downregulated plasma metabolites in preterminal samples compared to day (d): d 1, d 13, and d 25 samples.

				*p*-Value		
Compound	Comparison	FC	Log2(FC)	WSR Test	MWU Test	CID Fragments
LysoPA (18:2)	d 1 vs. preterminal	0.17627	−2.50414114	0.01	<0.001	433.2368 152.9961
	d 13 vs. preterminal	0.25821	−1.95338322	0.02	0.004	
	d 25 vs. preterminal	0.33539	−1.57608842	0.01	<0.001	
PE (22:6/18:2)	d 1 vs. preterminal	0.56565	−0.82201844	0.04	0.026	786.5251 327.2357 283.2443 279.2334
	d 13 vs. preterminal	0.47415	−1.07658456	0.02	0.025	
	d 25 vs. preterminal	0.67753	−0.56164327	0.35	0.002	
Androsterone	d 1 vs. preterminal	0.52337	−0.93409687	0.01	0.001	291.1971 249.1860 233.1534 203.1080
	d 13 vs. preterminal	0.65955	−0.60044606	0.038	0.04	
	d 25 vs. preterminal	0.69581	−0.52323	0.067	0.095	
Testosterone	d 1 vs. preterminal	0.62021	−0.68917131	0.114	0.018	281.6678 272.6580 162.9414 140.5447 104.1045 281.6606 272.6562 162.9465 148.5327 140.5424 104.1091
	d 13 vs. preterminal	0.54735	−0.86946444	0.019	0.01	
	d 25 vs. preterminal	0.64174	−0.63993919	0.114	0.025	

FC: fold change, WSR: Wilcoxon Signed-Rank test, MWU: Mann–Whitney U test.

## Data Availability

All relevant data are within the manuscript and its Supporting Information files.

## Supplementary Materials

The following supporting information can be downloaded at: https://www.mdpi.com/article/10.3390/metabo14010018/s1. Figure S1. A summary of key metabolites that participate in various metabolic pathways when comparing day 1 to the pre-irradiation timepoint. Figure S2. A summary of key metabolites that participate in various metabolic pathways when comparing day 13 to the pre-irradiation timepoint. Figure S3. A summary of key metabolites that participate in various metabolic pathways when comparing day 25 to the pre-irradiation timepoint. Figure S4. A summary of key metabolites that participate in various metabolic pathways when comparing day 1 to the preterminal timepoint. Figure S5. A summary of key metabolites that participate in various metabolic pathways when comparing day 13 to the preterminal timepoint. Figure S6. A summary of key metabolites that participate in various metabolic pathways when comparing day 25 to the preterminal timepoint. Table S1. Unpaired statistical summary comparing day (d) 1, d 13, and d 25 samples to pre-irradiation or preterminal samples. Table S2. Paired statistical summary comparing day (d) 1, d 13, and d 25 samples to preterminal samples. Table S3. A list of the notable metabolites (presented in KEGG ID format) and their related metabolic pathways present in the day 1 vs. pre-irradiation comparison. Table S4. A list of the notable metabolites (presented in KEGG ID format) and related metabolic pathways present in the day 13 vs. pre-irradiation comparison. Table S5. A list of the notable metabolites (presented in KEGG ID format) and related metabolic pathways present in the day 25 vs. pre-irradiation comparison. Table S6. A list of the notable metabolites (presented in KEGG ID format) and their related metabolic pathways present in the day 1 vs. preterminal comparison. Table S7. A list of the notable metabolites (presented in KEGG ID format) and related metabolic pathways present in the day 13 vs. preterminal comparison. Table S8. A list of the notable metabolites (presented in KEGG ID format) and related metabolic pathways present in the day 25 vs. preterminal comparison.

## References

[1] Health and Human Services HHS Enhances Nation’s Health Preparedness for Radiological Threats. https://www.hhs.gov/about/news/2016/10/06/hhs-enhances-nation-s-health-preparedness-radiological-threats.html.

[2] Gosden C., Gardener D. (2005). Weapons of mass destruction–threats and responses. BMJ.

[3] Gale R.P., Armitage J.O., Hashmi S.K. (2021). Emergency response to radiological and nuclear accidents and incidents. Br. J. Haematol..

[4] Gale R.P., Baranov A. (2011). If the unlikely becomes likely: Medical response to nuclear accidents. Bull. At. Sci..

[5] Gusev I.A., Guskova A.K., Mettler F.A. (2001). Medical Management of Radiation Accidents.

[6] Cohen J. (2011). Biodefense: 10 years after reinventing Project BioShield. Science.

[7] Russell P.K. (2007). Project BioShield: What it is, why it is needed, and its accomplishments so far. Clin. Infect. Dis. Off. Publ. Infect. Dis. Soc. Am..

[8] U.S. Department of Health and Human Services Project BioShield Annual Report, January 2014–December 2014. https://www.medicalcountermeasures.gov/media/36816/pbs-report-2014.pdf.

[9] Hall E.J., Giaccia A.J. (2012). Radiobiology for the Radiologist.

[10] DiCarlo A.L., Maher C., Hick J.L., Hanfling D., Dainiak N., Chao N., Bader J.L., Coleman C.N., Weinstock D.M. (2011). Radiation injury after a nuclear detonation: Medical consequences and the need for scarce resources allocation. Disaster Med. Public Health Prep..

[11] Waselenko J.K., MacVittie T.J., Blakely W.F., Pesik N., Wiley A.L., Dickerson W.E., Tsu H., Confer D.L., Coleman C.N., Seed T. (2004). Medical management of the acute radiation syndrome: Recommendations of the Strategic National Stockpile Radiation Working Group. Ann. Intern. Med..

[12] U.S. Food and Drug Administration Guidance Document: Product Development under the Animal Rule. http://www.fda.gov/downloads/drugs/guidancecomplianceregulatoryinformation/guidances/ucm399217.pdf.

[13] Singh V.K., Newman V.L., Romaine P.L., Hauer-Jensen M., Pollard H.B. (2016). Use of biomarkers for assessing radiation injury and efficacy of countermeasures. Expert Rev. Mol. Diagn..

[14] Coy S.L., Cheema A.K., Tyburski J.B., Laiakis E.C., Collins S.P., Fornace A. (2011). Radiation metabolomics and its potential in biodosimetry. Int. J. Radiat. Biol..

[15] Singh V.K., Seed T.M., Cheema A.K. (2021). Metabolomics-based predictive biomarkers of radiation injury and countermeasure efficacy: Current status and future perspectives. Expert Rev. Mol. Diagn..

[16] Pannkuk E.L., Fornace A.J., Laiakis E.C. (2017). Metabolomic applications in radiation biodosimetry: Exploring radiation effects through small molecules. Int. J. Radiat. Biol..

[17] Jefferson R.D., Goans R.E., Blain P.G., Thomas S.H. (2009). Diagnosis and treatment of polonium poisoning. Clin. Toxicol..

[18] Harrison J., Fell T., Leggett R., Lloyd D., Puncher M., Youngman M. (2017). The polonium-210 poisoning of Mr Alexander Litvinenko. J. Radiol. Prot. Off. J. Soc. Radiol. Prot..

[19] Nathwani A.C., Down J.F., Goldstone J., Yassin J., Dargan P.I., Virchis A., Gent N., Lloyd D., Harrison J.D. (2016). Polonium-210 poisoning: A first-hand account. Lancet.

[20] International Atomic Energy Agency The Radiological Accident in Goiânia. http://www-pub.iaea.org/MTCD/Publications/PDF/Pub815_web.pdf.

[21] Da Silva F.C., Hunt J.G., Ramalho A.T., Crispim V.R. (2005). Dose reconstruction of a Brazilian industrial gamma radiography partial-body overexposure case. J. Radiol. Prot. Off. J. Soc. Radiol. Prot..

[22] Singh V.K., Olabisi A.O. (2017). Nonhuman primates as models for the discovery and development of radiation countermeasures. Expert Opin. Drug Discov..

[23] Schule S., Gluzman-Poltorak Z., Vainstein V., Basile L.A., Haimerl M., Stroszczynski C., Majewski M., Schwanke D., Port M., Abend M. (2023). Gene Expression Changes in a Prefinal Health Stage of Lethally Irradiated Male and Female Rhesus Macaques. Radiat. Res..

[24] National Research Council of the National Academy of Sciences (2011). Guide for the Care and Use of Laboratory Animals.

[25] Singh V.K., Fatanmi O.O., Wise S.Y., Carpenter A.D., Olsen C.H. (2022). Determination of lethality curve for cobalt-60 gamma-radiation source in rhesus macaques using subject-based supportive care. Radiat. Res..

[26] Li Y., Singh J., Varghese R., Zhang Y., Fatanmi O.O., Cheema A.K., Singh V.K. (2021). Transcriptome of rhesus macaque (*Macaca mulatta*) exposed to total-body irradiation. Sci. Rep..

[27] Li Y., Girgis M., Wise S.Y., Fatanmi O.O., Seed T.M., Maniar M., Cheema A.K., Singh V.K. (2021). Analysis of the metabolomic profile in serum of irradiated nonhuman primates treated with Ex-Rad, a radiation countermeasure. Sci. Rep..

[28] Phipps A.J., Bergmann J.N., Albrecht M.T., Singh V.K., Homer M.J. (2022). Model for evaluating antimicrobial therapy to prevent life-threatening bacterial infections following exposure to a medically significant radiation dose. Antimicrob. Agents Chemother..

[29] Cheema A.K., Li Y., Moulton J., Girgis M., Wise S.Y., Carpenter A., Fatanmi O.O., Singh V.K. (2022). Identification of novel biomarkers for acute radiation injury using multiomics approach and nonhuman primate model. Int. J. Radiat. Oncol. Biol. Phys..

[30] American Veterinary Medical Association AVMA Guidelines for the Euthanasia of Animals: 2020 Edition. https://www.avma.org/sites/default/files/2020-01/2020-Euthanasia-Final-1-17-20.pdf.

[31] Singh V.K., Kulkarni S., Fatanmi O.O., Wise S.Y., Newman V.L., Romaine P.L., Hendrickson H., Gulani J., Ghosh S.P., Kumar K.S. (2016). Radioprotective efficacy of gamma-tocotrienol in nonhuman primates. Radiat. Res..

[32] Li Y., Girgis M., Jayatilake M., Serebrenik A.A., Cheema A.K., Kaytor M.D., Singh V.K. (2022). Pharmacokinetic and metabolomic studies with a BIO 300 oral powder formulation in nonhuman primates. Sci. Rep..

[33] Cheema A.K., Li Y., Singh J., Johnson R., Girgis M., Wise S.Y., Fatanmi O.O., Kaytor M.D., Singh V.K. (2021). Microbiome study in irradiated mice treated with BIO 300, a promising radiation countermeasure. Anim. Microbiome.

[34] Menon S.S., Uppal M., Randhawa S., Cheema M.S., Aghdam N., Usala R.L., Ghosh S.P., Cheema A.K., Dritschilo A. (2016). Radiation metabolomics: Current status and future directions. Front. Oncol..

[35] Crook A., De Lima Leite A., Payne T., Bhinderwala F., Woods J., Singh V.K., Powers R. (2021). Radiation exposure induces cross-species temporal metabolic changes that are mitigated in mice by amifostine. Sci. Rep..

[36] Cheema A.K., Li Y., Girgis M., Jayatilake M., Fatanmi O.O., Wise S.Y., Seed T.M., Singh V.K. (2020). Alterations in tissue metabolite profiles with amifostine-prophylaxed mice exposed to gamma radiation. Metabolites.

[37] Cheema A.K., Li Y., Girgis M., Jayatilake M., Simas M., Wise S.Y., Olabisi A.O., Seed T.M., Singh V.K. (2019). Metabolomic studies in tissues of mice treated with amifostine and exposed to gamma-radiation. Sci. Rep..

[38] Cheema A.K., Hinzman C.P., Mehta K.Y., Hanlon B.K., Garcia M., Fatanmi O.O., Singh V.K. (2018). Plasma derived exosomal biomarkers of exposure to ionizing radiation in nonhuman primates. Int. J. Mol. Sci..

[39] Pannkuk E.L., Laiakis E.C., Garcia M., Fornace A.J., Singh V.K. (2018). Nonhuman primates with acute radiation syndrome: Results from a global serum metabolomics study after 7.2 Gy total-body irradiation. Radiat. Res..

[40] Pannkuk E.L., Laiakis E.C., Singh V.K., Fornace A.J. (2017). Lipidomic signatures of nonhuman primates with radiation-induced hematopoietic syndrome. Sci. Rep..

[41] Cheema A.K., Mehta K.Y., Rajagopal M.U., Wise S.Y., Fatanmi O.O., Singh V.K. (2019). Metabolomic studies of tissue injury in nonhuman primates exposed to gamma-radiation. Int. J. Mol. Sci..

[42] Carpenter A.D., Li Y., Fatanmi O.O., Wise S.Y., Petrus S.A., Janocha B.L., Cheema A.K., Singh V.K. Metabolomic profiles in tissues of nonhuman primates exposed to total- or partial-body radiation. Radiat. Res..

[43] Cheema A.K., Mehta K.Y., Santiago P.T., Fatanmi O.O., Kaytor M.D., Singh V.K. (2019). Pharmacokinetic and metabolomic studies with BIO 300, a nanosuspension of genistein, in a nonhuman primate model. Int. J. Mol. Sci..

[44] Pannkuk E.L., Laiakis E.C., Fornace A.J., Fatanmi O.O., Singh V.K. (2018). A metabolomic serum signature from nonhuman primates treated with a radiation countermeasure, gamma-tocotrienol, and exposed to ionizing radiation. Health Phys..

[45] Cheema A.K., Mehta K.Y., Fatanmi O.O., Wise S.Y., Hinzman C.P., Wolff J., Singh V.K. (2018). A Metabolomic and lipidomic serum signature from nonhuman primates administered with a promising radiation countermeasure, gamma-tocotrienol. Int. J. Mol. Sci..

[46] Maan K., Baghel R., Dhariwal S., Sharma A., Bakhshi R., Rana P. (2023). Metabolomics and transcriptomics based multi-omics integration reveals radiation-induced altered pathway networking and underlying mechanism. NPJ Syst. Biol. Appl..

[47] Upadhyay M., Rajagopal M., Gill K., Li Y., Bansal S., Sridharan V., Tyburski J.B., Boerma M., Cheema A.K. (2020). Identification of plasma lipidome changes associated with low dose spacetType radiation exposure in a murine model. Metabolites.

[48] Pannkuk E.L., Laiakis E.C., Mak T.D., Astarita G., Authier S., Wong K., Fornace A.J. (2016). A lipidomic and metabolomic serum signature from nonhuman primates exposed to ionizing radiation. Metabolomics.

