# Supplementary material for: Metabolomic Changes in Plasma of Preterminal Stage of Rhesus Nonhuman Primates Exposed to Lethal Dose of Radiation
Source: Metabolites. 2023 Dec 27;14(1):18. doi: 10.3390/metabo14010018 (PMC10819041; doi:10.3390/metabo14010018)
Supplement: Supplementary file 1 [file metabolites-14-00018-s001.zip › 2023-12-21 Supplementary Figures.pptx]

## Slide 1
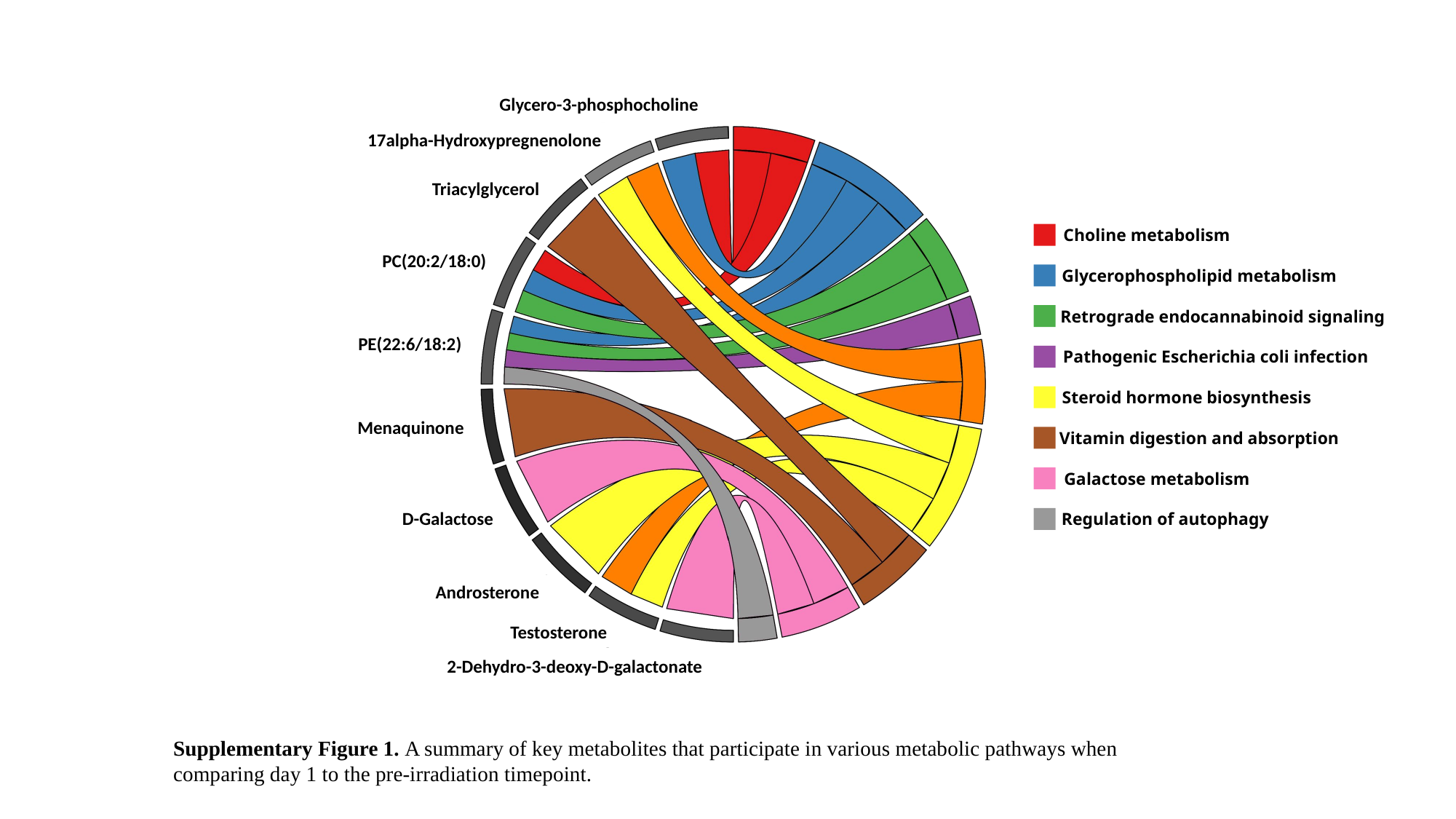

Glycero-3-phosphocholine
17alpha-Hydroxypregnenolone
Triacylglycerol
PC(20:2/18:0)
PE(22:6/18:2)
Menaquinone
D-Galactose
Androsterone
Testosterone
2-Dehydro-3-deoxy-D-galactonate
Choline metabolism
Glycerophospholipid metabolism
Retrograde endocannabinoid signaling
Pathogenic Escherichia coli infection
Steroid hormone biosynthesis
Vitamin digestion and absorption
Galactose metabolism
Regulation of autophagy
Supplementary Figure 1. A summary of key metabolites that participate in various metabolic pathways when comparing day 1 to the pre-irradiation timepoint.

## Slide 2
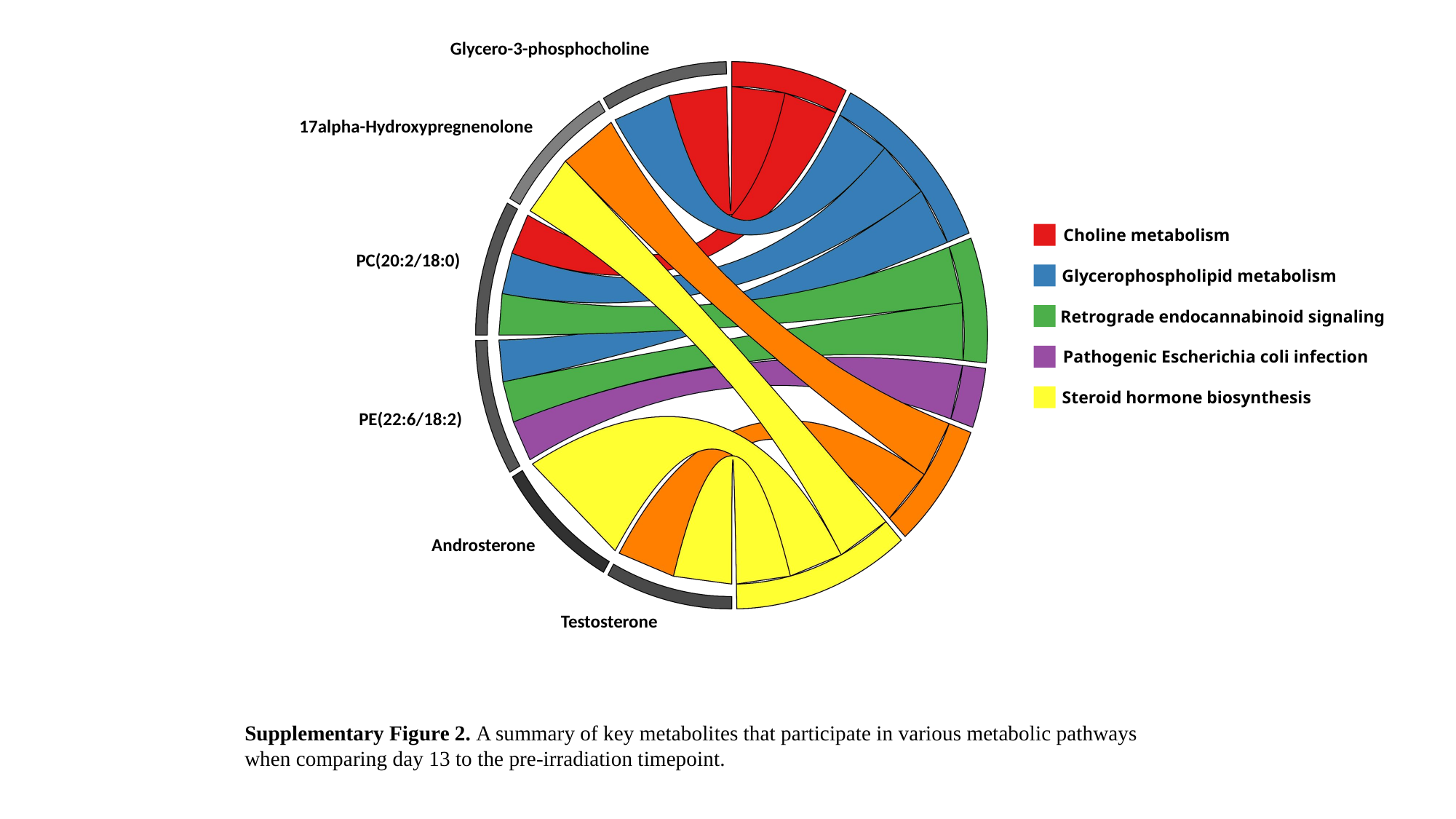

Glycero-3-phosphocholine
17alpha-Hydroxypregnenolone
PC(20:2/18:0)
PE(22:6/18:2)
Androsterone
Testosterone
Choline metabolism
Glycerophospholipid metabolism
Retrograde endocannabinoid signaling
Pathogenic Escherichia coli infection
Steroid hormone biosynthesis
Supplementary Figure 2. A summary of key metabolites that participate in various metabolic pathways when comparing day 13 to the pre-irradiation timepoint.

## Slide 3
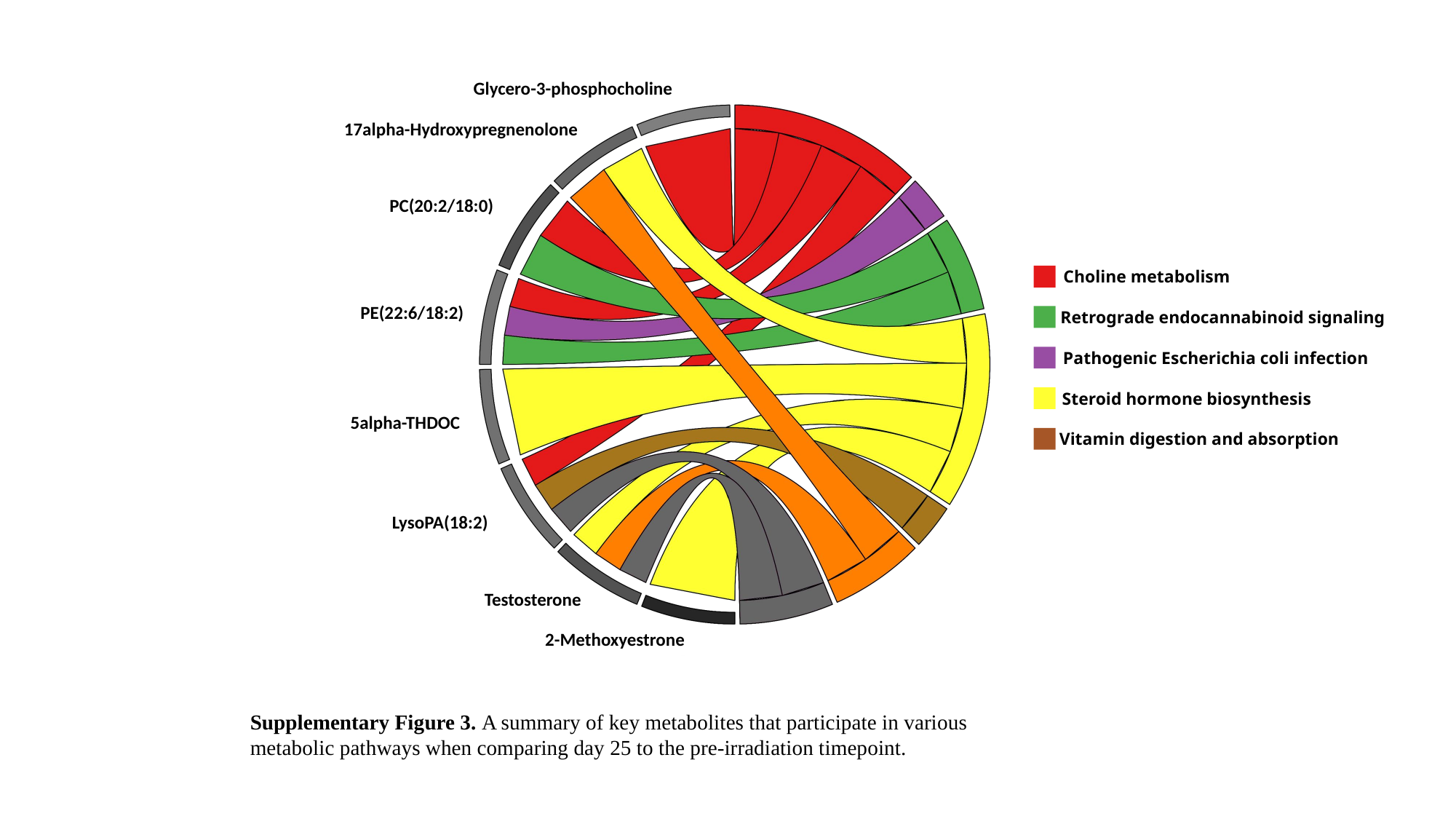

Glycero-3-phosphocholine
17alpha-Hydroxypregnenolone
PC(20:2/18:0)
PE(22:6/18:2)
5alpha-THDOC
LysoPA(18:2)
Testosterone
2-Methoxyestrone
Choline metabolism
Retrograde endocannabinoid signaling
Pathogenic Escherichia coli infection
Steroid hormone biosynthesis
Vitamin digestion and absorption
Supplementary Figure 3. A summary of key metabolites that participate in various metabolic pathways when comparing day 25 to the pre-irradiation timepoint.

## Slide 4
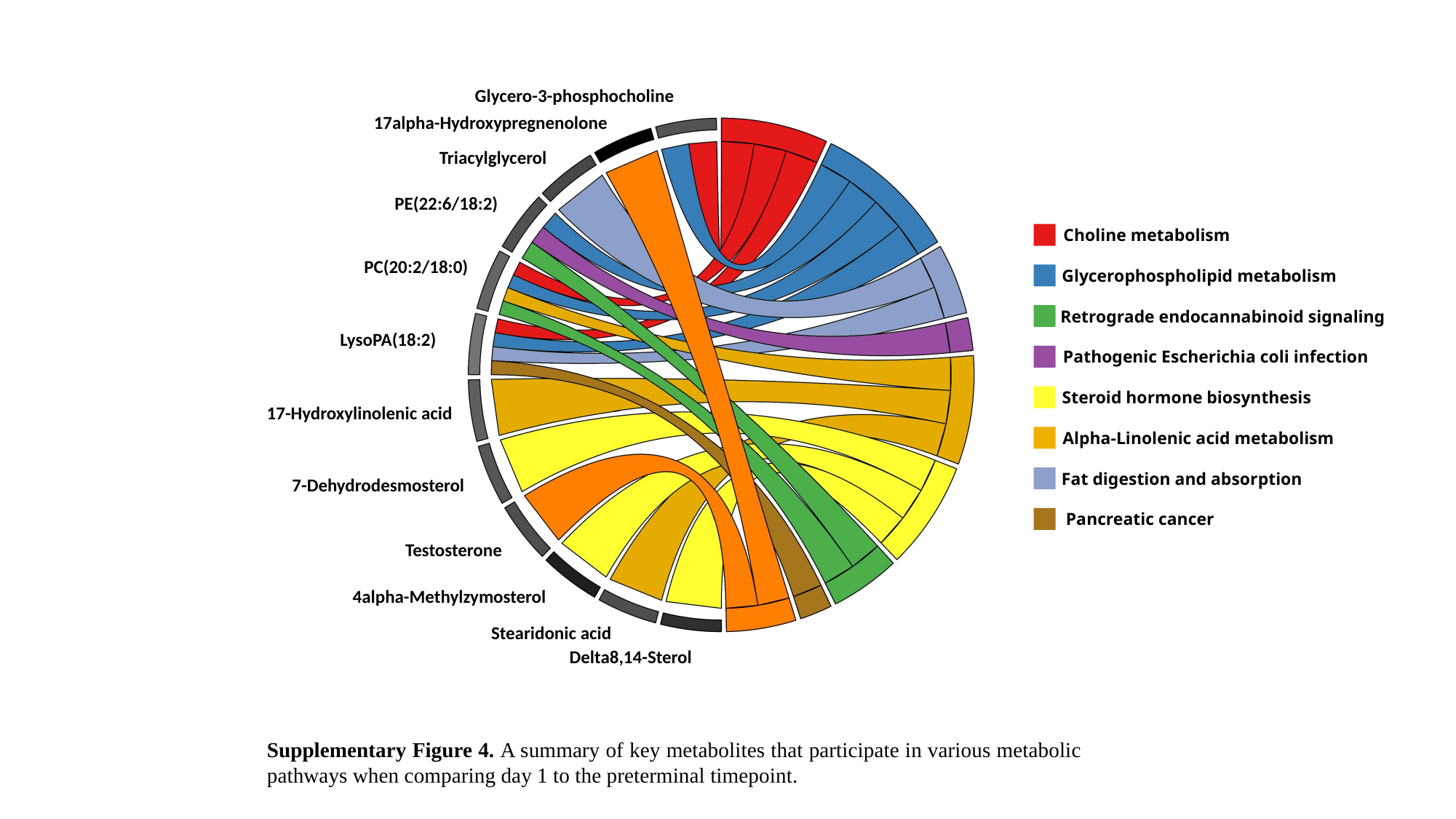

Glycero-3-phosphocholine
17alpha-Hydroxypregnenolone
Triacylglycerol
PE(22:6/18:2)
PC(20:2/18:0)
LysoPA(18:2)
17-Hydroxylinolenic acid
7-Dehydrodesmosterol
Testosterone
4alpha-Methylzymosterol
Stearidonic acid
Delta8,14-Sterol
Choline metabolism
Glycerophospholipid metabolism
Retrograde endocannabinoid signaling
Pathogenic Escherichia coli infection
Steroid hormone biosynthesis
Alpha-Linolenic acid metabolism
Fat digestion and absorption
Pancreatic cancer
Supplementary Figure 4. A summary of key metabolites that participate in various metabolic pathways when comparing day 1 to the preterminal timepoint.

## Slide 5
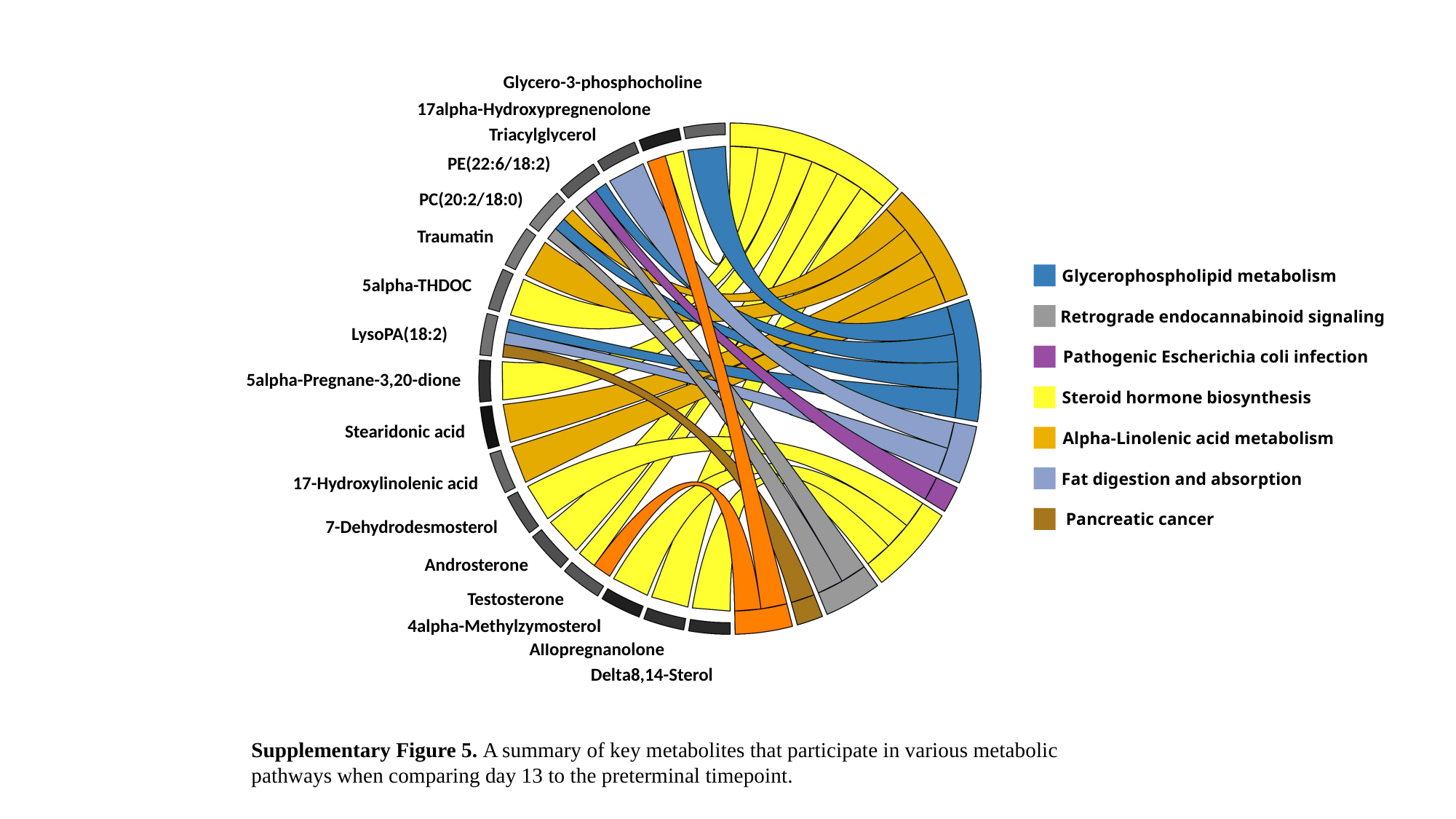

Glycero-3-phosphocholine
17alpha-Hydroxypregnenolone
Triacylglycerol
PE(22:6/18:2)
PC(20:2/18:0)
Traumatin
5alpha-THDOC
LysoPA(18:2)
5alpha-Pregnane-3,20-dione
Stearidonic acid
17-Hydroxylinolenic acid
7-Dehydrodesmosterol
Androsterone
Testosterone
4alpha-Methylzymosterol
Allopregnanolone
Delta8,14-Sterol
Glycerophospholipid metabolism
Retrograde endocannabinoid signaling
Pathogenic Escherichia coli infection
Steroid hormone biosynthesis
Alpha-Linolenic acid metabolism
Fat digestion and absorption
Pancreatic cancer
Supplementary Figure 5. A summary of key metabolites that participate in various metabolic pathways when comparing day 13 to the preterminal timepoint.

## Slide 6
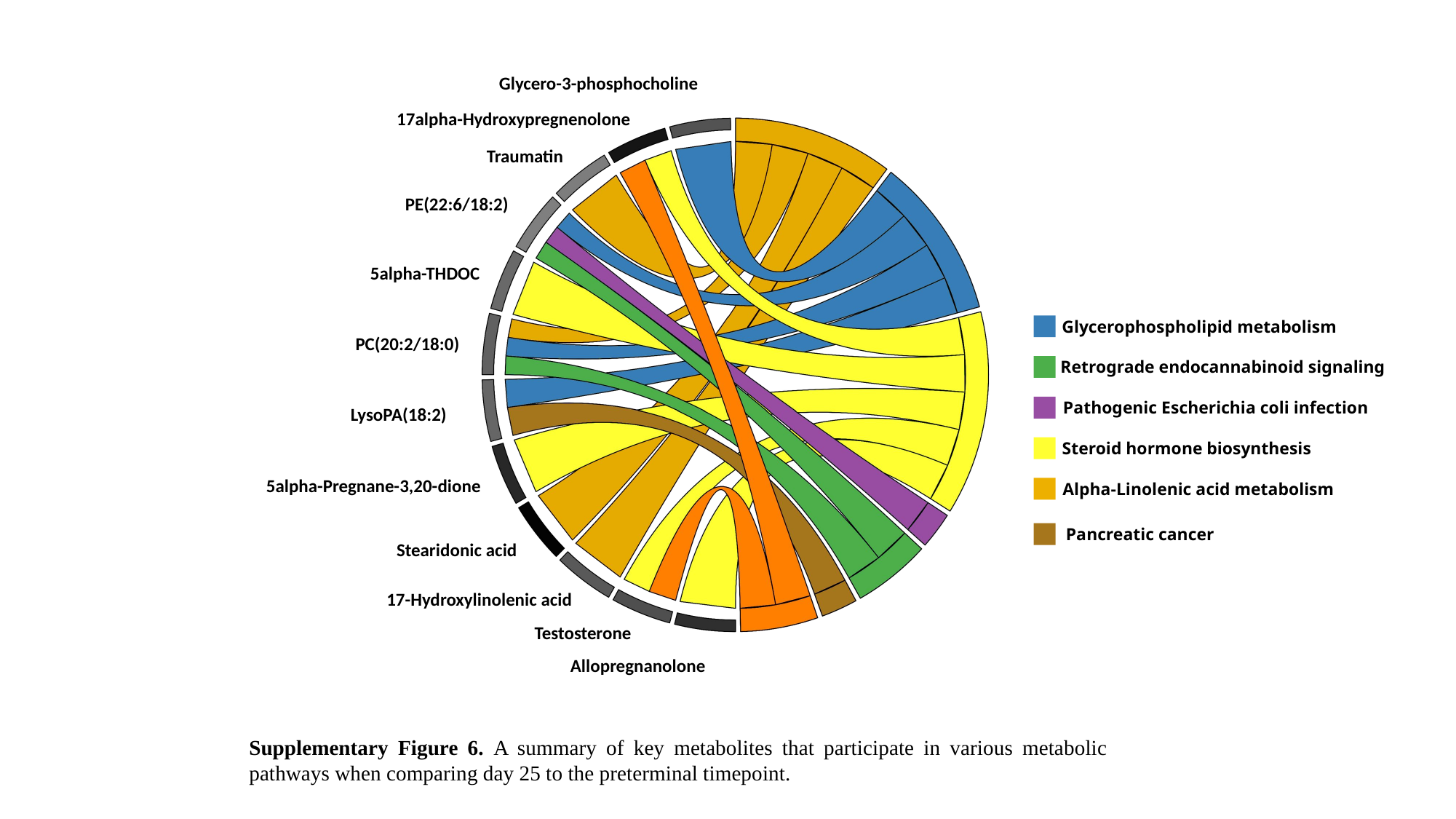

Glycero-3-phosphocholine
17alpha-Hydroxypregnenolone
Traumatin
PE(22:6/18:2)
5alpha-THDOC
Glycerophospholipid metabolism
PC(20:2/18:0)
Retrograde endocannabinoid signaling
Pathogenic Escherichia coli infection
LysoPA(18:2)
Steroid hormone biosynthesis
5alpha-Pregnane-3,20-dione
Alpha-Linolenic acid metabolism
Pancreatic cancer
Stearidonic acid
17-Hydroxylinolenic acid
Testosterone
Allopregnanolone
Supplementary Figure 6. A summary of key metabolites that participate in various metabolic pathways when comparing day 25 to the preterminal timepoint.
